# Dr. Henry Downer Ingraham

**Published:** 1904-06

**Authors:** 


Dr. Henry Downer Ingraham, of Buffalo, died at his residence, 405 Franklin street, May 23, 1904, after a long illness. This information was received while these columns were going through the press. An appropriate memorial will appear in the next issue of the Journal.

